# Postdischarge Impact of C-L Psychiatry Treatment in Obstetrical Inpatients

**DOI:** 10.5402/2011/456012

**Published:** 2011-07-24

**Authors:** Eileen Patricia Sloan, Sharon Kirsh

**Affiliations:** ^1^Perinatal Mental Health Unit, Department of Psychiatry, Room 928, Mount Sinai Hospital, 600 University Avenue, Toronto, ON, Canada M5G 1X5; ^2^Perinatal Mental Health Unit, Department of Psychiatry, Room 915, Mount Sinai Hospital, 600 University Avenue, Toronto, ON, Canada M5G 1X5

## Abstract

*Purpose*. Twenty-eight women, referred to C-L Psychiatry during their obstetrical inpatient stay were interviewed six months post-discharge to determine how they experienced the consultation process, whether they recollected and adhered to treatment recommendations, and whether they developed or had a recurrence of mental health problems post-discharge. *Method*. Semi-structured telephone interviews were conducted by a psychologist who had not been involved with patient care. *Results*. There was strong congruence between reason for referral as stated in psychiatric consult notes and participants' recollections and strong congruence and compliance regarding treatment recommendations. Sixty-four percent of women had concerns regarding mood post-discharge, of whom 66% sought professional help within six months. Participants' recommendations for improving the effectiveness of the C-L service to obstetrical inpatients pertained mainly to sensitivity to patients' feelings, consistency of message and personnel, and post-discharge follow-up. *Conclusions*. Obstetrical patients had good recollection of their experience of C-L psychiatry, and post-discharge compliance with treatment recommendations was high. A post-discharge telephone call might further enhance treatment compliance and encourage women who are struggling with mood difficulties to seek help. Contact between C-L psychiatry and patients' primary care physician may also enhance care post-discharge.

## 1. Introduction

Most obstetrical inpatient stays are short—often no more than one or two days—and the involvement of the inpatient consultation-liaison (C-L) psychiatry service, when required, is necessarily brief. Regardless of its compendious quality, however, its impact can have significance for the patient. 

We have previously reported on the characteristics of obstetrical inpatients referred for assessment by the C-L psychiatry service at Mount Sinai Hospital in Toronto [1]. Typically, C-L services do not follow up with patients after discharge. Borus et al. [2] argue that studies of C-L psychiatry's impact ought to be longitudinal and include examination of readmissions, persistence of physical and emotional dysfunction after hospital, adherence to prescribed medical regimens and rehabilitation programs, disability, and lost work days. While there is an extensive international literature on liaison mental health services, Callaghan et al. [3], in their review of 17 evaluative studies of these services, point out that most investigations examine the structure and process of liaison work, with little attention paid to *outcome* variables such as compliance with treatment recommendations and patient satisfaction. Furthermore, even in those instances where outcome is considered, patients' views tend not to be solicited. Where patients' views are sought, they tend to be elicited through satisfaction surveys. For example, Phillips et al. [4] surveyed former patients and the professionals who had referred them to a C-L psychiatry service in an obstetric-gynecology hospital. Mail-in questionnaires examined a number of variables, such as referral source and patient satisfaction with the consultation, and while their data provide a valuable contribution, the complexities of viewpoints are obscured through this forum. 

In a small number of studies, *qualitative* methods have been employed to garner patient evaluations of C-L psychiatry services and compliance with treatment recommendations. For example, an investigation by Rigatelli et al. [5] explores the effectiveness of a general hospital's C-L service; telephone interviews with 95 former inpatients and their primary care physicians 3–5 months after discharge indicated that 66% of patients reported high satisfaction with the psychiatric consultation, 22% were indifferent, and 11% expressed negative feelings. As for postdischarge compliance to treatment suggestions, the most common of which was psychopharmacology alone (68%), more than half of patients reported having filled their prescriptions and taken their medication, and half of these noted a positive outcome, a finding generally confirmed by their primary care physicians. These physicians, who had received a copy of the C-L consult note, agreed with the C-L psychiatric diagnosis in 81% of the study cases, and in 60% of cases, these primary care physicians assessed their patients' postdischarge conditions as improved. Rigatelli et al. conclude that “follow-up studies on outcome of psychiatric consultations are few and further analysis is strongly recommended.” 

A comparable study by Eales et al. [6] evaluated a C-L psychiatry service in an accident and emergency department and a general hospital setting. Service users, who had all presented with a psychiatric complaint, emphasized the necessity of excellent communication skills on the part of service providers; from the patients' perspective, this translates as feeling believed/listened to/taken seriously, receiving useful information, being responded to in a calming manner, receiving a novel perspective on their problems, and having an opportunity to talk without being interrupted by questions, especially during the beginning of the assessment. With regard to consultation outcome, some patients had felt involved in formulating decisions about their future and recalled having been told to return to the hospital if needed, while others had felt excluded from decision-making. The majority of patients felt the service could be improved if postdischarge followup was offered. Eales et al. conclude that it is important to engage service users in action planning to implement follow-up measures. 

In the interests of program evaluation and the development of best practice principles, we sought to ascertain the ongoing impact of our inpatient C-L Psychiatry service on the mental health of obstetrical inpatients after discharge, for example, whether they recollected and adhered to treatment recommendations made during their time in hospital and whether they developed or had a recurrence of mental health problems. We also wanted to understand from a phenomenological perspective patients' experience of the inpatient psychiatric consultation and to determine how the service could be rendered more effective.

## 2. Method

Consent for the study was obtained from the Mount Sinai Hospital Research Ethics Board. Psychiatric consult notes were reviewed for all obstetrical inpatients referred to the C-L Psychiatry service at Mount Sinai Hospital, Toronto, Canada, during a six-month period from September 2007 to February 2008. Each of these patients was contacted by letter six months after discharge to invite participation in a semistructured, 30–60 minute telephone interview. The letter was immediately followed up by a phone call to set a date for the interview [1]. 

Characteristics of the hospital and the C-L service are detailed in [1]. To summarize, Mount Sinai Hospital is a tertiary-level university-affiliated general hospital that cares for a large number of low-risk and high-risk pregnant women; there are approximately 7,000 deliveries per year. It is estimated that 40% of the hospital's obstetrical patients are “high-risk”, and it is not uncommon for high-risk antenatal patients to be transferred from smaller and/or remote communities in Ontario or even from other provinces in Canada.

Data collected during the telephone interview included demographic information (age, relationship status, employment, level of education, ethnic or racial identification, religious affiliation, number of years of residency in Canada); reproductive history; participants' *recollections *of reason for referral (RFR) to psychiatry, diagnosis, treatment recommendations, psychotropic medications taken at the time of, or initiated as a result of, the consultation, and recommendations for postdischarge care/followup; participants' reasons for noncompliance with treatment recommendations; participants' evaluation of the inpatient psychiatry consultation; participants' recommendations for improving the service. 

The areas explored here include (1) congruence between data derived from consultation notes and participants' recollections pertaining to RFR, diagnosis, and treatment recommendations; (2) participants' postdischarge compliance with recommendations; (3) participants' concerns about mood after discharge; (4) participants' perceptions of the benefits and their critical assessments of the inpatient psychiatric consultation process; (5) participants' recommendations for improvement to the process, both inhospital and followup.

## 3. Results

Between September 2007 and February 2008, 62 obstetrical inpatients were referred to the C-L Psychiatry service at Mount Sinai Hospital. Of these, 28 women (45%) agreed to participate in the current study; eight women could not be reached because they had moved and there was no forwarding address (in four of these cases there was “no fixed address”); in nine cases the phone number was no longer in service; four women who were contacted chose not to participate; five patient files were not accessible; eight women consistently did not answer their phone, and in the interests of privacy and confidentiality, a message was not left.

Of the 28 study participants, 21% were antenatal inpatients at the time of the psychiatry consultation while 79% were postpartum. Seventy-one percent (71%) of patients reported a past psychiatric history. Thirty-six percent (36%) of the sample currently had a normal delivery but were psychiatrically symptomatic and endorsed a past psychiatric history. Sixty-one percent (61%) of the total sample were experiencing an adverse perinatal event (e.g., premature birth, termination for fetal anomaly, unexpected Down Syndrome, preeclampsia, traumatic delivery); 55% of the total sample had had a previous adverse reproductive event (e.g., spontaneous abortion, termination of pregnancy for fetal anomaly, extreme prematurity). 

Demographic data for participants are shown in Table 1. Most notable is educational level: 71% had a university or college degree, and another 25% had attended university or college for at least one year. At six-month postpartum, 11% of participants had returned to the workforce, while 32% intended to remain at home as full-time homemakers. Most women (86%) were raised in Canada, and the majority reported their ethnic and/or religious affiliation as “None” (36%) or Jewish (21%). Over half (54%) of participants were primigravidae.

### 3.1. Congruence between Patients' Recollections and C-L Psychiatry Notes

**(a) Reason for Referral**

In 79% of cases, women's perception of the RFR was congruent with the RFR stated in the psychiatry consult note. While terminology used by patients differed from that of the psychiatrist or psychiatric resident, the essence of their reasons was usually concordant, for example, consult note states: “High risk for PPD; crying, overwhelmed” and the patient describes her RFR this way: “I have a history of major depression, and on day one postpartum the emotions came fast and furious.”

In some cases, contributory factors most pressing for the patient may not have been deemed so by the obstetrical or psychiatric staff. For example, a woman (GA 27 weeks) suffering from placenta previa had been airlifted to the hospital from a town several hundred miles away. One month after admission, while on antenatal bedrest, she was referred to the C-L psychiatry service. The RFR reported in the consult note was: “History PPD; currently mildly anxious; twins; planned C-section and hysterectomy; patient worried about her health”. During the study interview at six months post-discharge, the patient recalled the reasons for her referral as a history of PPD, but more important were the extreme loneliness she had been experiencing in the total absence of supports in Toronto, her strong feelings of insecurity about her relationship with her husband and other children, and her intense fear of dying in childbirth. From her perspective, the RFR was multifactorial, the very least of which was her past history of PPD.

In five cases there was a marked discrepancy between the stated RFR and the woman's perceived reason for referral. For example, RFR stated in the consult note: “High anxiety, mood lability; acute stress response following high-risk (HELLP) premature C-section” versus the patient's perceived RFR: “The social worker suggested the consult because my baby was premature and I was very sick, but I wasn't at all anxious, just sad to be separated from my other child.”

Two women reported being asymptomatic from a psychiatric perspective at the time of their consult.

Participants were asked to recall who had made the referral to Psychiatry. Approximately 25% did not know—a psychiatry resident and/or staff psychiatrist simply arrived at the bedside to do a consultation. In 11% of cases, the woman recalled that family members or she herself had asked for a referral. A member of the nursing staff was thought to have been instrumental in 29% of cases, and similarly there was a recollection of 29% involvement of either the antenatal or postpartum social worker. Physicians were less likely to be recalled as having made the referral (21%).

**(b) Diagnosis**

Study participants were asked to recall whether they had been given a psychiatric diagnosis by the inpatient C-L service and whether they could recall the specific diagnosis: 61% of women could not recall being given a diagnosis; whereas 64% were assigned a DSM-IV-R diagnosis according to the consult note. Hence, 25% of the sample was unaware that a diagnosis had been made. Where women *did* recall, there was a very high degree of congruence with the diagnosis noted in the chart.

**(c) Treatment**

The recommendations for treatment made by the C-L Psychiatry service fell into three, nonmutually exclusive categories (see Table 2) (i) Participation in the “5-day/5-night programme” whereby women remain in hospital for 5 days and 5 nights, while neonates are cared for in the nursery overnight to allow new mothers as much consolidated sleep as possible [7]; (ii) postdischarge treatment; (iii) initiation or continuation of psychotropic medication.

Twelve women were offered the 5-day/5-night programme, and 10 women accepted.

With regard to postdischarge treatment, at six months there was complete congruence between recommendations articulated in consult notes and participants' recall of these. Women accurately remembered whether C-L psychiatry had suggested contacting the C-L psychiatrist on a per needed outpatient basis (46%), whether the hospital had organized outpatient psychiatric followup prior to discharge (7%), whether followup with the preadmission mental health professional was recommended (14%), or whether no follow-up recommendation was made (25%).

Sixty percent (*n* = 17) of the sample left the hospital with a prescription for psychotropic medication which had either been started or continued in hospital (see Table 3). There was an almost perfect congruence between medications noted in patients' charts and their specific recollections six months after discharge.

### 3.2. Compliance with Recommendations for Postdischarge Management

Where postdischarge psychiatric care had been suggested (*n* = 21), 81% of participants followed through by making an appointment; the majority (71%) of these women did so within two months after leaving the hospital. Hence, more than half (54%) of the total sample sought outpatient psychiatric or psychotherapeutic care within eight weeks of leaving the hospital.

Among the 19% (*n* = 5) who did not comply with recommendations for treatment after discharge, women articulated a variety of reasons.

I'm still really depressed about a lot of things, but I'm stubborn, and I like to help myself through soul-searching.

I figured I'd eventually get over it (termination of pregnancy for fetal anomaly). I can't indulge myself; if I go down that path, I'll never get out of it. I felt myself slipping into depression in the hospital, and I hit rock bottom, so I know I can only go up from there. I didn't want a crutch. I wanted to get better.

I don't like to leave the baby for long periods and I live fairly far from the hospital. After I go back to work, I'll contact the Psychiatry department.

Regarding compliance with recommendations for psychotropic medication (*n* = 17), 70% filled the prescription and took the medication as suggested; almost all (91%) of these women had previous experience with psychotropic medication. Twelve percent filled the prescription but stopped taking the medication without consulting their doctor; 18% chose not to fill their prescription, citing the following reasons: worried about side effects on baby; disliked taking medication; felt she did not need medication.

### 3.3. Concerns Regarding Mood after Discharge

When participants were asked whether they had had any concerns about their mood after discharge, 64% (*n* = 18) reported at least *some* concern. These fell into four categories.

**(a) Depression and/or Generalized Anxiety**

I'm extremely resentful, angry, and depressed. My husband works from 4:30 pm until 2:00 am, and my marriage is disintegrating because of it. I want him home for the bewitching hour, at 5:30.

I'm monitoring my mood and slowly decreasing my medication. My mood is stable now. I try to keep busy. I get out all the time; otherwise, I'd be very low. Depression can consume you if you don't have people around. I have money and resources, but what about all those women who don't? The first few months were the hardest time I've ever had to deal with.

**(b) Grief Following Fetal/Neonatal Demise or having a Child with an Unexpected Congenital Condition**

I still feel a lot of sadness, anger, grieving for the baby I thought I'd have.

I'm not as depressed as at the beginning. I wouldn't have been ready to go back to work at four months. Maybe now, but I'm not sure. I'm not as motivated in general as I want to be.

“I went through the “why me?” I went home with nothing. By now, I'm all worried out.”

**(c) Anxiety Expressed as Heightened Concern for Health of Neonate/Infant**

I was a goner when I came home, crying all day long. I was up every hour-and-a-half. I'm still worried that something will happen to the baby—SIDS—so there's a breathing monitor in her crib. But I'm still looking over there all night, making sure she's breathing.

I'm still anxious, but doing better. I feel I can continue with (psychiatrist). She said I had postpartum anxiety, but was heading toward postpartum depression. It felt like terror. For me it's harder than for most people to keep “normal” in perspective, so if the baby gets sick, I might find it harder to cope.

**(d) Insomnia**

Sleeplessness and a lack of bonding were freaking me out. He's a very good baby, but I was so sensitive to his sounds. I was upset with him, upset with me. … I don't have enough money to hire a regular babysitter for relief during the day.

Among women reporting some mood concerns, three participants (11%) reported developing depression after discharge, and five (18%) reported a continuation of prepregnancy anxiety.

I'm feeling guilty about my decision (to terminate for fetal anomaly), although I know it was the right decision. I'm crying all the time. My family is very supportive, but they don't know our secret. … I'm going to call one of the psychiatrists at (the hospital). I'm concerned about my mood. Last week I saw my family doctor. I have no patience with the kids, I'm crying, anxious, and I can't get the image of the fetus out of my head. I'm always wondering what the fetus would have, or could have, been. My husband holds it together for my sake, he's very supportive, but he breaks down occasionally. My doctor thinks I might have postpartum depression. … I have to go on because of my other children, but I often feel like curling up in a ball.

I thought I was just being a normal mother, having temper tantrums, but my mother said this wasn't normal and that I needed pills. My family doctor said I have psychomotor depression and that's worse than postpartum depression. I have to be a mommy AND a nurse (baby born with renal failure, on dialysis, awaiting transplant). I used to sweat and have panic attacks when the baby cried, but now I'm more confident as a mother, and my boyfriend and I don't fight as much. The Cipralex makes me calmer and tolerant and easy-going. It makes me want to get out and do something, not just wallow.

A significant proportion (66%) of women with post-discharge mood concerns were seeing either a mental health professional or their family physician with regard to this issue. Of concern is that 34% had not yet sought help, 17% intending to do so in the near future, and 17% not intending to do so at all.

On the positive side, 36% of participants reported a relatively smooth postpartum transition, with no mood concerns; for example,

I have down days, but I'm doing fine. No panic attacks since discharge. I felt different with this birth, no sadness. I was more prepared. The first pregnancy came too quickly after marriage, I was later in age than most mothers with my first, and I had to adjust from being single and independent to being a mother.

“My mood is really good, I'm coping well. We're thriving!”

**Summary of Findings Regarding Congruence and Compliance:**

- *reason for referral*: 79% congruence between report in consult notes and participants' recollections,
- *diagnosis*: 61% recalled no diagnosis given by the C-L psychiatrist (36% of consult notes report no diagnosis); excellent congruence between diagnosis noted and recalled,
- *recommendation for postdischarge therapy: *75% of sample recalled the suggestion to seek therapy; strong congruence between report in consult note and participants' recollections regarding specific mental health professional (e.g., hospital psychiatrist, family doctor, mental health professional seen prior to pregnancy) to contact after discharge,
- *compliance regarding postdischarge therapy*: of women recalling the suggestion for postdischarge therapy, 81% made an appointment and saw either a mental health professional or a family physician, and of these, 71% (i.e., 54% of total sample) saw such a professional within two months after discharge; 19% chose not to seek therapeutic help,
- *recommendation for psychotropic medication*: 60% of participants reported having received a prescription before leaving hospital; this is congruent with the percentage reported in consult notes,
- *compliance regarding psychotropic medication*: of participants receiving prescriptions, 70% filled their prescription and took their medications as directed by the C-L psychiatrist (91% of these women had previous experience with psychotropic medications); 12% filled their prescription and stopped taking their medication without consulting their physician; 18% did not fill their prescription,
- *mood concerns after discharge*: 64% of the sample reported being concerned about their mood within the six months after discharge; 36% reported no such concerns,
- *decision to seek help regarding concerns*: of those women who expressed specific concerns about their mood after discharge, 66% sought help (44% from mental health professional; 22% from family doctor); 17% intend to seek help in the near future; 17% did not intend to seek help even though they had concerns,
- *referral source*: 25% could not recall referral source; 29% recalled nurse, 29% recalled social worker on the obstetrical unit, 21% recalled physician as referral source.

### 3.4. Perceived Benefits and Criticisms of C-L Psychiatry Service

**(a) Benefits**

The majority (75%) of participants reported finding the psychiatry consultation helpful in at least one of three ways.

**(i) Emotional Support/Empathy:**

At first I hesitated to have a consultation. I'm not crazy! But when it was explained to me, I embraced it. I can't say enough about it, both nursing and psychiatry. They were so interested in seeing me healthy. … You don't feel abandoned and alone. In some places, they just stick you in a room and forget about you.

I didn't have to hide my feelings; I didn't have to be strong. Nobody tried to say I was wrong to feel what I felt. You feel like a failure if it (pregnancy) doesn't work ‘cause it's your God-given right as a woman to have a baby.

It was nice to have someone listen to you, and give you resources in case you needed them. It's a resource for support and help.

I was in physical pain (post C-section) and paranoid. I had the sense that somebody cares—the nurses and psychiatrist and resident came often. The psychiatrists were respectful and they asked the right amount of questions.

**(ii) Initiation/Resumption of Psychotropic Medications.**

I felt relieved to have (C-L psychiatrist) confirm that I was on the right track by returning to Effexor (venlafaxine).

It was extremely helpful because it helped me identify that I needed help. (The C-L psychiatrists) didn't know my history and I didn't want to bring another person into the mix, but they were a conduit to (my regular psychiatrist). They agreed to contact him and he told them to give me a prescription for Paxil. So they were treating symptoms, not root causes. … Social work and psychiatry worked as an integrated team.

**(iii) 5-Day/5-Night Programme:**

This programme was recommended for 12 women (43%) in our sample, most of whom had either a psychiatric history such as PPD, postpartum psychosis, or bipolar disorder, or were experiencing a particularly low mood and were without adequate social supports. Several women who participated in the programme stated unequivocally that they had not been in shape psychologically to leave the hospital in the usual timely fashion (i.e., after 1-2 days), and the 5-day/5-night programme afforded them time, emotional support, and practical help with their newborn:

I was in tears, and (C-L psychiatrist) talked me through it. She recommended the 5-day/5-night programme. I wanted to go home, but was accepting of her advice…. The psychiatrist and social worker worked together, talking with my husband and mom to formulate a plan to help support me. The programme was extremely helpful. It worked for me because I spoke out and this brought a lot of people together, working as a team: the psychiatrist, the resident, social worker, nurses, my family doctor. They discussed what was in my best interest. I was very glad to have it.

The programme was so helpful. I felt totally taken care of. Someone came everyday from psychiatry, and showed up even over the holiday. The psychiatry resident wasn't a doctor; he said, “Your baby is beautiful”. He was human. Everyone from psychiatry was kind, compassionate, listened well; they were very practical. The human part was so important to me—they were human.

Just before we left, I saw (the C-L psychiatrist) again and her resident, and she discussed my Prozac and made certain I had supports in place back home. The programme was wonderful, knowing I could nap when I needed and was close enough to see the baby.

I turned down the 5/5 programme because I was feeling anxious about leaving my two-year-old at home. But when I got home, I realized I'd made a mistake. I felt my husband and mom were mad at me for being sad, and so they weren't supportive.

**(b) Criticisms of the C-L Service**

Satisfaction with the service provided by C-L Psychiatry was generally high, but there were aspects that women felt needed to be reconsidered or improved upon. One participant reported feeling “judged” by psychiatry; other women found that the consultation exacerbated their sad or negative feelings:

Talking makes me think more about my problems. I didn't want to cry any more. I'm comfortable with my family doctor; she's not judging me, but I feel psychiatrists judge you.

When the psychiatrist and resident asked for my history (severe PPD 7 years earlier during which patient vigorously shook baby), I experienced a lot of guilt. I felt they were digging. I know I was sick in the past, but I hate to think about what I did to my first child. Going over the details made me feel guilty. I felt vulnerable and embarrassed in front of the resident and my husband and the psychiatrist—a whole crowd.

I got very upset by the psychiatrist's questions because he was asking me about my stillbirth. I told the nurse after, I explained why I was so upset. When the psychiatrist came back and I explained and after that I felt a bit more comfortable. Then he was understanding and sympathetic.

It was hard to open up to people I didn't know. Then it felt weird to leave the hospital, and I was always afraid that someone might think badly of me, that I was a bad mother because I was anxious and afraid to go home.

Some women reported feeling not completely listened to/understood:

The psychiatrist should be aware of the signs when a person needs a soft place, and not be clinical. I could have used a softer touch, someone who could've laughed with me. I wanted to put *him* at ease. If a person cracks a joke, the psychiatrist should try to go with it.

Some women who remained in hospital as part of the 5-day/5-night programme reported that they received mixed messages from nurses and/or lactation consultants; this barrage of confusing information/opinion was perceived as insensitivity to the woman's needs:

The lactation consultants pressured me to wake up at night; only one of them acknowledged that I could both breast and bottle feed. If a mother is having a nervous breakdown, then you need to change the rules. It didn't help my depression to be pressured and pushed.

I felt relieved after my initial consultation with (C-L psychiatrist) … she arranged for me to get sleep, but the nurses seemed unaware of this option of having the baby sleep in the nursery—for 3 nights they took the baby for an hour or two, but they didn't seem to know the alternate arrangement was possible.

### 3.5. Patients' Recommendations for Improvements to the C-L Psychiatry Service

When asked how the C-L Psychiatry service could be improved, participants offered recommendations that fall into six categories.

**(a) Time-Related**

Have the psychiatry resident schedule an appointment with the new mother rather than showing-up unannounced.

Set appointment times with patients rather than dropping-by.

Have the woman stay an extra day (after termination) and then have the psychiatric consult. I would have been more receptive to information on the third day. I felt so rushed after the fetus was delivered; I needed time to recover.

They (psychiatrist and residents) stayed too long, the three of them, for well over an hour.

**(b) Consistency of Personnel**

I would've liked more consistency in the people I saw (on bedrest one month, followed by termination). I had to repeat my story so many times, for each resident and psychiatrist. You want to feel like you're important, that it's not just their job, that you're not a lab rat. They need to build trust. I was guarded sometimes because of all this changeover.

They should talk to the existing (external) psychiatrist to get the patient's history, and then come in more prepared. Otherwise they make you feel like a textbook case.

**(c) Sensitivity to What Patient Feels Her Needs are**

First, ask if a girl wants to talk about it, or whether she's just asking for a sleeping aid. Don't drill into a person. Speak in a conversational, not a doctor, way. Don't say, “How does that make you feel?” Wow, I didn't realize psychiatrists actually say that, and not just on TV! It made me feel so awkward. So I told the psychiatrist what she wanted to hear because I was exhausted and just wanted the damn sleeping pills.

A psychiatrist ought to be part of the team, along with the geneticist, social worker, and nurse, that tells the patient about bad news.

**(d) Group Support for Antenatal Women in Hospital for Extended Periods**

A formal support group should be offered to inpatients to share whatever they're going through.

I would have appreciated a group session while I was an inpatient. The lactation course draws tons of women. It would be helpful to have an opportunity to talk about feelings with other women.

**(e) Consistency of Message between Disciplines**

Lactation consultants should be educated about the 5/5 programme: “breast is best” might be true, but the rules need to bend for 5/5 patients.

Patients should be made aware that they're included in the 5/5 programme so they understand why the nurses want to take their baby away.

**(f) Postdischarge Follow-Up**

When study participants were asked for suggestions regarding postdischarge followup, 92% strongly recommended a telephone call to check up on mental health status postpartum and to provide an offer of help where needed/requested. Although women differed in opinion as to timing (anywhere from 1 to 6 weeks postpartum), each stressed the importance of such a contact.

A follow-up call from the hospital would take the pressure off the woman to take the initiative. I would've come in for an appointment, to keep the relationship with (the C-L psychiatrist), but it takes a lot of effort to find the number and make the call, but I would've come in if someone had called me to make an appointment.

Do a follow-up call ideally within the first week. If (the woman) doesn't need it, then fine. Do just what you're doing now—how are you doing? Getting enough support? Getting out?—so much emphasis is placed on the baby, but not much attention is paid to the mom postpartum.

Follow-up is an excellent idea after one week. Check in with the patient, offer resource information, and mainly offer validation to women. You're the only one I've told how I felt about coming home, that I didn't think how I felt was normal.

Do follow-up calls at one month and so on to check on women…. After a baby is born, the sense of community is gone. You feel alone, like an immigrant. Motherhood is like being a new immigrant; you walk down the street and you're all alone.

There shouldn't be too long a gap between discharge and (follow-up); on the other hand, the timing shouldn't be too short because a woman needs time to digest because she's so darned overwhelmed at first. It would have been helpful to have a psychiatrist come to the house shortly after returning home. Also, continuity of care is important—it meant a lot to me to be able to see (the same C-L psychiatrist) both in the hospital and after discharge.

I just felt dismissed once we left (the hospital). I recommend a follow-up call at one month, not before, because women are just coming out of baby blues and realizing what's what and whether they need some help.

One woman offered a dissenting view:

I would not have liked a call because it would have made me feel like I must have a problem if someone is checking on me.

## 4. Discussion

The current study, set in the obstetrical unit of a tertiary-level, university-affiliated general hospital, provides understanding of the C-L psychiatry consultation process from the perspective of the patient herself. We achieved a participation rate of 45% of all former obstetrical inpatients who had received a consultation during the six-month study period. This response rate is comparable with that reported by others [4, 5, 8]. We acknowledge that our findings are likely biased by the fact that our participants are relatively high functioning, the majority having a university or college qualification. A significant proportion of women could not be included in the study because they had no fixed address or telephone contact number at the time of the consultation. It is possible that these women would be less likely to participate even if we had been able to contact them, and their recall of the consultation with C-L Psychiatry may have been dissimilar to that of the women interviewed. Furthermore, willingness to engage with recommendations for postdischarge care might have been lower.

Within the group that did participate, compliance to treatment recommendations, both psychotherapeutic and psychopharmacological, was high. The extent to which compliance is buttressed by family physicians is unclear. We have not been in the practice of sending a copy of the consult note or postdischarge recommendations to the family physician. Rigatelli et al. [5] stress the importance of this step, arguing that the consultation is essentially useless in the absence of followup, since its effects are then limited to the short hospital stay. Given what we know of the impact of postpartum mental illness, and the fact that the involvement of the C-L psychiatrist is usually very brief, contact with family physicians should be considered crucial. Of the women who had concerns regarding mood after discharge, 34% had not yet (at 6 months after discharge) sought help. Bearing in mind that our group was quite highly educated and that considerable effort is made to highlight the importance of postpartum mental health, this figure is concerning. Had their family physicians been made aware of the assessment and recommendations by C-L Psychiatry, the figure might not have been as high.

In general, study participants perceived their interaction with our C-L Psychiatry service in a positive light. A significant proportion did, however, refer to at least one way in which the interaction could be improved. For example, some women had no idea who initiated the consultation—a psychiatrist simply appeared at the bedside without forewarning or the patient's approval. This echoes Phillips et al. [4] who noted that patients were often unaware of the reason for a referral to Psychiatry and/or what was to be achieved from the consultation. 

The most pressing issue among our participants was the perceived absence of postdischarge followup (this would have been suggested/arranged where deemed necessary). Some women felt “abandoned” by a system that worked hard to offer a show of concern, but turned its back after discharge, a time when support was most needed. This reflects the findings of Eales et al. [6] who noted that the majority of service users believed they should have been offered followup (a letter, telephone call, or follow-up clinic), and that any followup ought to include a reminder of the treatment plan. Mistiaen and Poot [9] have carried out a comprehensive review of studies which examined the impact of follow-up telephone calls by C-L psychiatry services in the first month after discharge. They found that while no adverse effects were reported, studies show clinically equivalent results between telephone follow-up groups and control groups. Whether or not this holds true for the postpartum population remains to be determined. The feedback received in the current study suggests that postdischarge calls would be perceived as supportive and might encourage some women to seek care in a more timely fashion. The limiting factor is lack of personnel. However, if it can be determined that this is a cost-effective and preventative measure for the postpartum population, a case could be made for ensuring staff (e.g., psychiatric nursing) availability as an essential part of a comprehensive C-L service. 

As a result of this study we are now seeking to implement a number of changes on our C-L psychiatry service with obstetrical inpatients, namely,

- ensure patient understands why we were consulted, by whom, and what she can expect from the consultation,
- ensure that all disciplines involved in a patient's care are aware of the treatment plan—obstetrical staff, nursing, social work, and where appropriate, lactation consultants,
- send a discharge note to the patient's primary care physician and/or community psychiatrist,
- ensure postdischarge followup with mental health services where deemed appropriate,
- develop an inpatient group for supportive psychotherapy for antenatal women who are in hospital for prolonged periods,
- provide a follow-up call to all obstetrical C-L patients approximately two weeks after discharge.

## Figures and Tables

**Table 1:** Demographics of study participants (*N* = 28).

Age	Relationship	Education	Current employment status	Ethnic/racial affiliation	Religious affiliation	Years in Canada	Other living children
Mean age = 34	Married 75%	Completed college/university 71%	Parental leave 46%	“None” 36%	“None” 36%	Entire life 86%	None 54% 1–32% 2–10% 5–4%
	Common law 18%	Some college/university 25%	Full-time homemaker 32%	Jewish 21%	Jewish 21%	Philippines 1983; Zimbabwe 1993; Iran 2000; Pakistan 2005	
	Single 7%	High school 4%	Part-time job 7%	“White” 11%	Roman catholic 18%		
			Seeking employment 7%	4% each: Black, “Black-and-White”, Greek orthodox Iranian, Italian, Pakistani, Philippine, Portuguese	7% each: Muslim, Greek orthodox, Christian		
			Full-time job 4%		Protestant 4%		
			Social assistance 4%				

**Table 2:** Treatment recommendations and compliance as reported by participants.

Categories of treatment suggested (*N* = 28)	Recommendations for postdischarge therapy followup	Patients' compliance with treatment recommendations (*n* = 21)	Time between discharge and first appointment with therapist (*n* = 17)
Inpatient 5-day/5-night programme: offered to 12, accepted by 10 (36%)	Patient to contact C-L psychiatrist if needed: 46% (*n* = 13)	Made appointment with psychiatrist/therapist: 81% of women for whom any followup was recommended	1 month or less: 47%

Postdischarge therapy followup (refer to next column)	Postdischarge appointment with psychiatrist arranged by hospital: 7% (*n* = 2)	Did not make appointment: 19% of women for whom any followup was recommended	1-2 months: 24%

Psychotropic medication (refer to Table 3)	Patient instructed to follow up with preadmission mental health provider: 14% (*n* = 4)		More than 4 months: 18%

	Postdischarge therapy strongly urged: 7% (*n* = 2)		

	No treatment recommendations made: 25% (*n* = 7)		No answer: 12%

**Table 3:** Psychotropic medications: recommendations and compliance as reported by participants.

Recommendations and compliance	% (*N* = 28)
Left hospital with psychotropic Rx	60% (*n* = 17)

Filled Rx and took medication	70% (91% of these had experience with psych drugs prior to hospital stay)

Filled Rx but stopped meds without medical consultation	12% (all had been on meds in recent past)

Did not fill Rx	18%

No psych medications administered in hospital or prescribed for postdischarge	40% (*n* = 11)
